# Cyclin-Dependent Kinase Inhibitors in Hematological Malignancies—Current Understanding, (Pre-)Clinical Application and Promising Approaches

**DOI:** 10.3390/cancers13102497

**Published:** 2021-05-20

**Authors:** Anna Richter, Nina Schoenwaelder, Sina Sender, Christian Junghanss, Claudia Maletzki

**Affiliations:** Department of Medicine, Clinic III—Hematology, Oncology, Palliative Medicine, Rostock University Medical Center, 18057 Rostock, Germany; Anna.Richter@med.uni-rostock.de (A.R.); Nina.Schoenwaelder@med.uni-rostock.de (N.S.); Sina.Sender@med.uni-rostock.de (S.S.); Christian.Junghanss@med.uni-rostock.de (C.J.)

**Keywords:** CDK4/6 inhibitors, predictive biomarker, mechanisms of resistance, pharmacological inhibition, combination strategies

## Abstract

**Simple Summary:**

Cyclin-dependent kinases are involved in the regulation of cancer-initiating processes like cell cycle progression, transcription, and DNA repair. In hematological neoplasms, these enzymes are often overexpressed, resulting in increased cell proliferation and cancer progression. Early (pre-)clinical data using cyclin-dependent kinase inhibitors are promising but identifying the right drug for each subgroup and patient is challenging. Certain chromosomal abnormalities and signaling molecule activities are considered as potential biomarkers. We therefore summarized relevant studies investigating cyclin-dependent kinase inhibitors in hematological malignancies and further discuss molecular mechanisms of resistance and other open questions.

**Abstract:**

Genetically altered stem or progenitor cells feature gross chromosomal abnormalities, inducing modified ability of self-renewal and abnormal hematopoiesis. Cyclin-dependent kinases (CDK) regulate cell cycle progression, transcription, DNA repair and are aberrantly expressed in hematopoietic malignancies. Incorporation of CDK inhibitors (CDKIs) into the existing therapeutic regimens therefore constitutes a promising strategy. However, the complex molecular heterogeneity and different clinical presentation is challenging for selecting the right target and defining the ideal combination to mediate long-term disease control. Preclinical and early clinical data suggest that specific CDKIs have activity in selected patients, dependent on the existing rearrangements and mutations, potentially acting as biomarkers. Indeed, CDK6, expressed in hematopoietic cells, is a direct target of MLL fusion proteins often observed in acute leukemia and thus contributes to leukemogenesis. The high frequency of aberrancies in the retinoblastoma pathway additionally warrants application of CDKIs in hematopoietic neoplasms. In this review, we describe the preclinical and clinical advances recently made in the use of CDKIs. These include the FDA-approved CDK4/6 inhibitors, traditional and novel pan-CDKIs, as well as dual kinase inhibitors. We additionally provide an overview on molecular mechanisms of response vs. resistance and discuss open questions.

## 1. Introduction

Despite the clinical implementation of novel treatments like kinase inhibitors, hypomethylating agents or pathway modulators during the last decades, several hematological malignancies still face a poor prognosis. Relapsed or refractory courses are particularly difficult to manage, often exhibiting multiple drug resistances and intimidating survival rates [1,2,3,4]. The FDA approval of the cyclin dependent kinase (CDK) 4/6 inhibitors (CDKIs) palbociclib, ribociclib, and abemaciclib for hormone receptor positive and human epidermal growth factor receptor 2 negative locally advanced or metastatic breast cancer treatment led to numerous promising clinical studies investigating CDKIs in solid neoplasms. This provides a rationale for the emerging intensive testing in hematological malignancies, raising hopes for improving patients’ prognoses. Given their wide spectrum of cellular modulation including cell cycle control, transcription, DNA repair, epigenetic regulation, proliferation, and apoptosis, CDKs represent promising molecular targets for leukemia and lymphoma treatment [5].

Cell cycle regulation is a key mechanism to prevent malignant cell proliferation and uncontrolled cell division. Aleem and Arceci reviewed the roles of CDKs in controlling cell cycle and development of hematological malignancies in detail [6]. Here, we focus on present and future therapeutic approaches to overcome CDK-influenced cell cycle control malfunction (Table S1) and will thus only offer a short introduction on CDK pathways and their role on leukemogenesis. In brief, cell cycle control is mainly mediated by serine/threonine CDKs acting as catalytic subunit when activated by their respective cyclins. CDK activity is further modulated by physiological CDKIs and posttranslational modification, resulting in transcriptional regulation, DNA damage repair mechanisms, metabolism, or epigenetic processes. In addition to the “classical” CDKs directly influencing the cell cycle, further kinases act as indirect modulators to regulate transcription or epigenetic signaling [6].

Hematopoietic stem cells are relatively quiescent to prevent stem cell exhaustion [7,8,9]. Once these stem cells initiate cell cycle induction, the cells proliferate extensively to provide hematopoiesis [10]. Therefore, the cell cycle of hematopoietic stem cells must be controlled thoroughly. Dysregulation of CDKs and associated cyclins is frequently observed in hematological malignancies. CDK6 is predominantly expressed in hematopoietic cell types and loss of CDK6 results in impaired generation of several blood cell types. In contrast, overexpression and chromosomal translocation of CDK6 is observed in acute lymphoblastic leukemia (ALL) and lymphoma. CDK4/6 inhibition is achieved by physiological CDKI p16*^INK4A^*, the most frequently deleted locus in human cancer. Translocations of the *MLL* gene locus are common in acute myeloid leukemia (AML) and account for most infant ALL cases. CDK6 is a direct target of MLL fusion proteins and thus activated. Finally, CDK6 can be activated by *FLT3*-ITD-mediated down-regulation of cyclins D2 and D3. Besides CDK6, other CDKs are also involved in leukemogenesis. For example, the AML driver mutation *FLT3*-ITD is an activator of CDK1. Further, mutations and deletions of the cyclin C and CDK19 locus on 6q21 result in altered Notch1 regulation especially in T-ALL [6].

## 2. CDK4/6 Inhibitors

While the pan CDKI flavopiridol was the first CDK inhibitor applied clinically [11], recent CDKIs are more specific, with most drugs targeting only a subset of CDKs. CDK6 has a central role in hematopoiesis and CDK6-deficient mice show reduced production of erythrocytes, granulocytes, macrophages, neutrophils, and thrombocytes, as well as thymic atrophy [6]. Neutropenia is therefore the most common and dose-limiting adverse event in the clinical use of CDK6 inhibitors [12]. CDK6 is also a direct target of MLL fusion proteins which are common in AML and ALL [13,14]. This results in transcriptional activation of CDK6 and subsequent initiation of leukemic processes [14,15,16]. Further, CDK6 can be activated via *FLT3*-ITD-mediated upregulation of cyclins D2 and D3 [17]. In mantle cell lymphoma, the characteristic t(11;14) translocation induces ectopic cyclin D1 expression, also resulting in CDK6 and CDK4 upregulation [18]. In pediatric B-ALL, p16*^INK4A^* deletions, especially occurring during relapse, are a key feature of dysfunctional CDK4/6 control and an associated dismal prognosis [19].

### 2.1. Palbociclib

The CDK4/6 inhibitor palbociclib was widely evaluated in solid tumors and is now also analyzed in a variety of hematopoietic malignancies. It demonstrated significant pre-clinical in vitro and in vivo efficacy in AML cells with *FLT3*-ITD [17,20] and TKD mutations [21], *RUNX1/ETO* translocation [22] and *MLL* rearrangement [15,23]. In a recent study investigating de novo transformation of granulocyte/macrophage progenitor cells to AML, Chen et al. demonstrated that transient palbociclib application is capable of halting progenitor cell proliferation and preferentially abrogated the most proliferative progenitor cell subsets. Palbociclib further reduced the progenitor cell transformation in vivo, resulting in reduced AML burden and prolonged survival. This suggests that cell cycle inhibition decreases the likelihood of malignant transformation in vivo [24].

In T-ALL cells as well as B-ALL cells featuring *MLL* or BCR-ABL1 rearrangements, palbociclib controlled cell growth via G1 arrest and Rb dephosphorylation both in vitro and in vivo [14,25,26,27,28]. A phase I clinical trial investigating palbociclib in relapsed ALL children is currently underway.

Similar promising antitumoral effects were observed in multiple myeloma, anaplastic large-cell lymphoma, and mantle cell lymphoma, where palbociclib was also capable of overcoming ibrutinib resistance [29,30,31,32,33,34]. In a study with 17 relapsed mantle cell lymphoma patients, palbociclib achieved one complete remission (CR) and two partial responses (PR), five patients had a progression-free survival of at least one year with reduced tumor metabolism and proliferation [35]. A phase I study in non-Hodgkin lymphoma showed stable disease (SD) in one third of the participants and two out of 68 patients had PR [36].

Palbociclib was then evaluated in several combinations both preclinically and clinically. The combination with proteasome inhibitor bortezomib was effective in an immunocompetent myeloma mouse model. Inhibition of CDK4/6 by palbociclib induced G1 arrest and enhanced bortezomib susceptibility via increased mitochondrial depolarization [30]. This combination was also evaluated in a clinical phase I/II trial in refractory/relapsed (R/R) myeloma together with dexamethasone, demonstrating an overall response rate of 20% and SD in 44% of the participants [37]. A phase I trial in R/R mantle cell lymphoma with palbociclib and bortezomib achieved CR in one out of 19 patients [38]. Combined palbociclib and ibrutinib treatment has been evaluated in R/R mantle cell lymphoma. From 27 patients in this phase I study, 67% responded and 37% had CR [39].

**Figure 1:**
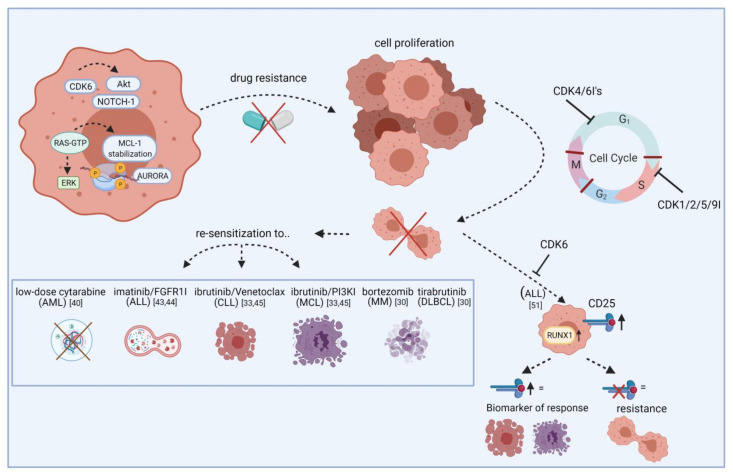
Re-sensitization of drug-resistant cells by CDK inhibitors. The scheme illustrates how CDKIs can conquer resistance of leukemic blasts towards targeted agents. Leukemic cells show frequent overexpression or constitutive activation of oncogenic kinases like AURORA and AKT. While these kinases mediate drug resistance via apoptosis inhibition or increased tumor cell proliferation, blocking specific CDKIs may help to make cells more vulnerable to certain approved drugs, including cytarabine, imatinib, and ibrutinib. This effect is likely mediated by cell cycle checkpoint blockade, resulting in impaired cell division and proliferation. Additionally, blocking CDKs induces CD25 abundance on ALL cells and this may act as a biomarker for response. Vice versa, lack of CD25 expression indicates resistance. FGFR1I, fibroblast growth factor receptor 1 inhibitor; PI3KI, phosphatidyl inositol 3 kinase inhibitor; MCL, mantle cell lymphoma; MM, multiple myeloma; DLBCL, diffuse large B-cell lymphoma. Created with BioRender.

Further combination partners are evaluated preclinically in vitro and in vivo. In AML, the cytarabine dose could be reduced after palbociclib priming (Figure 1) [40]. Besides cell cycle regulation, CDK6 also controls gene expression of oncogenic kinases by directly binding promoter sites. These include, among others, AURORA and AKT, both of which are known mediators of drug resistance. *FLT3* mutations can further contribute to pathway activation in AML. Combined palbociclib and pan-AURORA kinase inhibitor danusertib or AKT inhibitor MK-2206 treatment resulted in synergistic anti-leukemic effects in *FLT3*-ITD and TKD mutated AML cells [21].

Although there is a clear connection between BCR-ABL1 fusion and cell cycle regulation, only very limited data is available for the evaluation of CDKIs in chronic myeloid leukemia (CML). Rangatia and Bonnet have shown that lack of BCR-ABL1 leads to G1 phase arrest and a decrease in cyclin D1. Physiological CDKIs p21 and p27 subsequently exhibited increased gene expression [41]. Schneeweiss-Gleixner et al. recently reported that palbociclib synergizes with tyrosine kinase inhibitor ponatinib in BCR-ABL1*^T315I^* mutated CML, via reducing proliferation and inducing G1 arrest. This is of importance because tyrosine kinase inhibitor resistance is frequently observed in this CML subtype, leading to challenging clinical problems [42]. In ALL, palbociclib synergizes with fibroblast growth factor receptor 1 inhibitor PD-173074 and imatinib (Gleevec) [43,44]. Increased apoptosis rates compared to palbociclib mono application were achieved with PI3Kδ inhibitor GS-1101 and BET protein bromodomain antagonist JQ1 in mantle cell lymphoma [45,46]. In myeloma and diffuse large B-cell lymphoma, synergism was achieved with dexamethasone and Bruton’s tyrosine kinase inhibitor tirabrutinib [29,47].

**Figure 2:**
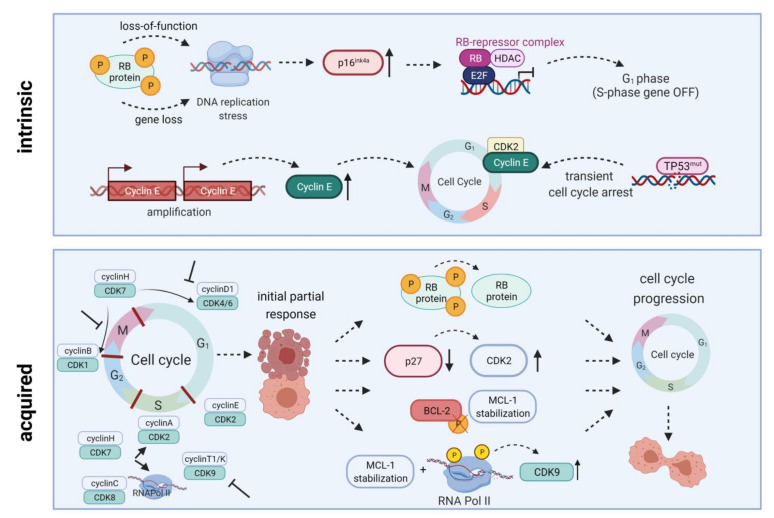
Intrinsic and acquired resistance mechanisms towards CDKIs. Intrinsic resistance is driven by lack of functional Rb protein, leading to DNA replication stress, high-level expression of endogenous CDK4/6 inhibitor p16*^INK4A^*, and inactivation of E2F-regulated genes. Another resistance mechanism is based on Cyclin E amplification and overexpression driving cell cycle progression. Quite in line, *TP53* mutations are generally associated with low response to CDK inhibition. Acquired resistance mechanisms include Rb loss, CDK2 reactivation upon p27 downregulation, a truncated BCL-2 protein lacking the phosphorylation loop domain, as well as prolonged MCL-1 stability along with RNA polymerase II phosphorylation and CDK9 kinase domain up-regulation. All these mechanisms may ultimately contribute to cell cycle progression and thus CDKI resistance. HDAC, histone deacetylase; E2F, E2F transcription factor family; RNA Pol II, RNA polymerase II. Created with BioRender.

To demonstrate their anti-leukemic potential, CDK4/6 inhibitors rely on expression of downstream signaling protein Rb. Intrinsic or acquired lack of Rb function results in resistance towards therapeutic CDK4/6 targeting [12]. Additionally, palbociclib treatment can lead to p27 downregulation, thus reactivating CDK2 and cell cycle progression. Transcription factor *FOXO3A* can additionally control *p27* gene expression. However, it was not influenced in palbociclib-resistant cells; ruling out the possibility of *FOXO3A* directly induced *p27* downregulation. Palbociclib-sensitive cell lines exhibited a similar drop in *p27* gene expression, while protein abundance remained stable. Hence, the *p27*-downregulating effect is rather due to posttranslational modification than reduced *p27* gene expression (Figure 2) [17].

### 2.2. Ribociclib

Ribociclib (LEE011) is another CDK4/6 inhibitor that demonstrated significant anti-proliferative and apoptosis-inducing effects in AML and B-ALL cell lines and primary samples, probably mediated via G1 arrest and senescence [48]. A recent study on pediatric B-ALL evaluated ribociclib in combination with dexamethasone. They found significant basal overexpression of CDK6 in B-ALL cells. Subsequent inhibition of CDK4/6 using ribociclib resulted in G1 phase arrest, reduced cell proliferation, and apoptosis induction accompanied by Rb dephosphorylation. Adding dexamethasone synergistically improved anti-neoplastic effects in B-ALL cell lines as well as primary samples [49].

Pikman et al. investigated ribociclib in *NOTCH1*-mutant and wildtype T-ALL and found both subtypes sensitive to the inhibitor. The combination with glucocorticoid dexamethasone and mTOR inhibitor everolimus acted synergistically in vitro and in vivo. In contrast, antagonistic effects were observed with several drugs used in T-ALL standard chemotherapy including methotrexate, mercaptopurine, asparaginase, or doxorubicin [50].

In T-ALL, CDK6 is required for AKT- or Notch1-induced leukemia initiation. Jena et al. demonstrated increased *CD25* and *RUNX1* expression upon CDK6 inhibition and that CD25 ablation results in T-ALL leukemogenesis. They further showed that CD25 mediates the therapeutic response to ribociclib, suggesting CD25 deletion as a potential mechanism of resistance to CDK6 inhibitors as well as predictive marker for ribociclib response (Figure 1) [51]. A clinical phase I study examined ribociclib in solid neoplasms and lymphoma and found SD in ten out of 70 patients [52].

### 2.3. Abemaciclib

While ribociclib was mainly analyzed in acute leukemias, abemaciclib (LY2835219) has been more widely evaluated in lymphoma. Here, inhibited proliferation and induced apoptosis was reported in germinal center-derived B-cell lymphoma cell lines, but not in activated B-cell like diffuse large B-cell lymphoma cell lines [53]. In a clinical trial, 71% of the participating R/R mantle cell lymphoma patients achieved SD and 36% had PR [54]. A subsequent phase II study with 28 patients reached CR in two patients and an overall response rate of 36% with an overall survival of 16 months [55]. Another clinical trial is currently evaluating abemaciclib with fulvestrant in advanced or metastatic solid tumors and lymphomas.

Further preclinical studies found abemaciclib effective in *MLL*-rearranged AML cell lines and xenografts [56] as well as myeloma cell lines [57]. Nakatani et al. recently identified that t(8;21) rearranged AML cell lines are more vulnerable to palbociclib and abemaciclib than non-t(8;21) AML cells [58]. Molecularly, this is due to higher cyclin D2 levels, a common response marker towards abemaciclib. In t(8;21) rearranged AML cells, abemaciclib induced the expected G1 arrest, leading to impaired cell proliferation and decreased MAPK and AKT pathway signaling. Another interesting finding of this study was autophagosome formation which significantly increased apoptosis induction upon combined application of abemaciclib and autophagy inhibitors [58].

### 2.4. Lerociclib

The novel CDK4/6 inhibitor lerociclib (G1T38) was capable of decreasing Rb phosphorylation and induced G1 cell cycle arrest in leukemia and lymphoma cell lines in vitro. Of note, lerociclib demonstrated a superior efficacy compared to palbociclib and did not induce severe neutropenia in an estrogen receptor positive breast cancer dog animal model, which is a common side effect of CDK4/6 inhibitors. Using mouse xenograft models, the authors further demonstrated that lerociclib accumulated within the tumor but not in plasma, implying less severe effects on myeloid progenitor cells compared to palbociclib therapy. Indeed, plasma concentrations after palbociclib treatment were significantly higher than the concentration needed to inhibit cell proliferation in several cell lines. In contrast, after lerociclib treatment, plasma concentrations dropped faster. The authors thus claim that palbociclib-induced neutropenia is a product of CDK4/6 inhibition in the bone marrow, preventing proliferation of healthy bone marrow cells [59].

CDK4/6 inhibitors including the novel promising molecule lerociclib are without doubt the most intensively researched group of CDKIs in both, solid and hematological malignancies. While palbociclib, ribociclib, and abemaciclib already obtained FDA approval for breast cancer, current preclinical and clinical studies for leukemia and lymphoma also focus on inhibitors targeting other CDKs.

## 3. CDK7/8/9 Inhibitors

**Figure 3:**
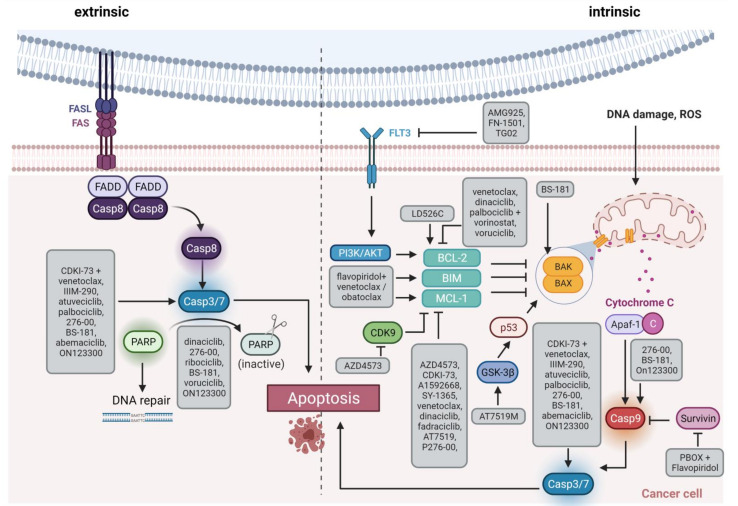
Schematic overview of apoptosis induction. The extrinsic pathway via Fas/FasL interaction is shown on the left side, and the intrinsic pathway via members of the BCL-2 family such as BAX/BAK on the right side. The highlighted boxes indicate the active form of the molecule. The gray boxes list therapeutic substances and how they influence apoptosis. FAS, Fas cell surface death receptor; FASL, FAS ligand; Casp, caspase, PARP, Poly(ADP ribose) Polymerase; FLT3, FMS related receptor tyrosine kinase 3; PI3K, phosphatidyl inositol 3 kinase; AKT, AKT serine/threonine kinase; ROS, reactive oxygen species; BCL-2, BCL2 apoptosis regulator; BIM, BCL2 like 11; MCL-1, MCL1 apoptosis regulator; BAK, BCL2 antagonist/killer 1; BAX, BCL2 associated X, apoptosis regulator; GSK-3β, glycogen synthase kinase 3-beta; p53, tumor protein 53; Apaf-1, Apoptotic peptidase activating factor 1. Created with BioRender.

CDKs 7, 8, and 9 are novel emerging targets in preclinical research. Indeed, recent data reveal anti-leukemic activity upon inhibition of these transcriptional regulators. CDK9 is a global transcriptional regulator and part of the super elongation complex controlling RNA polymerase II phosphorylation and elongation. AFF family members, which are frequently fused to *MLL* in acute leukemias, are also part of the super elongation complex, thus mis-localizing CDK9 to *HOX* gene promoters and inducing abnormal CDK9 expression, cell growth, and proliferation [60]. Another role for CDK9 is transcriptional regulation of MCL-1, it may thus influence intrinsic apoptosis induction (Figure 3) [61].

### 3.1. AZD4573

So far, AZD4573 is the only selective CDK9 inhibitor invested clinically in hematological malignancies. A preclinical study on different hematological neoplasms proved significant induction of apoptosis via MCL-1 suppression in vitro and in vivo [62]. The same study also evaluated the BCL-2 inhibitor venetoclax as combination partner to overcome treatment failure or leukemic progress after AZD4573 cessation. Both, MCL-1 and BCL-2 are anti-apoptotic members of the intrinsic apoptosis initiation cascade. Combined therapy with AZD4573 and venetoclax induced long-term inhibition of leukemic cell proliferation in all animals, even in models intrinsically resistant to either monotherapy (diffuse large B-cell lymphoma SU-DHL-4; AML model OCI-AML3) [62].

### 3.2. CDKI-73

The non-selective CDK9 inhibitor CDKI-73 proved anti-proliferative and pro-apoptotic capacity in chronic lymphoblastic leukemia (CLL) [63], diffuse large B-cell lymphoma [64], ALL and AML [65] cells, as well as in animal models. Walsby et al. evaluated the anti-neoplastic potential of CDKI-73 together with fludarabine in CLL and found decreased *MCL1* gene expression after CDK9 inhibition while fludarabine had the opposite effect. Combined application of CDKI-73 and fludarabine downregulated RNA polymerase II mediated genes *MCL1*, *XIAP*, *CCND1*, and *CCND2*. Of note, synergistic effects were also observed under CD40L-expressing pro-survival co-culture conditions with initial fludarabine resistance [63]. In diffuse large B-cell lymphoma, CDK9 inhibitors including CDKI-73 frequently induce histone 3 lysine 27 trimethylation, which is associated with tumor progression. A recent study therefore evaluated CDKI-73 with histone methyltransferase EZH2 inhibitors EPZ6438 and GSK126 and found synergistic anti-proliferative effects. The authors also identified drastically increased apoptosis and DNA damage in response to combined treatment [64]. The same group previously evaluated combinatorial effects of CDKI-73 with venetoclax in ALL and AML and elucidated synergistic induction of apoptosis via PARP and caspase 3 cleavage as well as XIAP downregulation [65]. Many more CDK9 inhibitors have been preclinically evaluated in a broad range of hematological malignancies [66,67,68,69,70,71].

### 3.3. CDK8 Inhibitors

CDK8 regulates transcription as part of the mediator complex or by phosphorylation of transcription factors [72]. In primary AML and ALL samples with BCR-ABL1 translocation, Menzl et al. found that mTOR signaling is usually deregulated in CDK8-deficient cells. They subsequently developed the small molecule YKL-06-101, targeting both CDK8 and mTOR, which had significant anti-leukemic potential in vitro and in vivo [73]. Another selective CDK8 inhibitor, SEM120, is effective in AML in vitro and in vivo and currently evaluated in a phase Ib clinical trial in AML and myelodysplastic syndrome [74].

### 3.4. CDK7 Inhibitors

First CDK7 inhibitors are also arising and are being tested in AML cell lines. The CDK7/12/13 inhibitor THZ1 showed anti-proliferative effects and induced apoptosis in a *RUNX1/ETO* rearranged cell line [75]. A preclinical study investigating the effect of THZ1 in myeloma revealed decreased cell proliferation and survival as well as RNA polymerase II, CDK 1, 2, and 9, MCL-1, BCL-XL, and c-MYC downregulation in vitro and in vivo. Addition of venetoclax or carfilzomib significantly increased the antitumor efficacy [76].

SY-1365, which is under clinical investigation for solid tumors, decreased MCL-1 protein levels and induced transcriptional changes mainly of oncogenic transcription factor genes and members of cell cycle and DNA damage repair related pathways. Of note, this inhibitor was more effective in cells with low BCL-XL expression. When combined with BCL-2 inhibitor venetoclax, antitumor effects were synergistically increased in vitro and in vivo [77]. Finally, BS-181 inhibited CDK7 and induced apoptosis in Jurkat T-ALL cells [78].

Collectively, CDK7/8/9 inhibitors hold promise for being implemented in trials because of their unique ability to modify transcriptional processes and regulate apoptosis. Other approaches featuring down-regulation of these kinases are less specific, resulting in a broader spectrum of mechanistic actions to be dealt with.

## 4. Pan CDK Inhibitors

### 4.1. Flavopiridol

Flavopiridol (alvocidib) is a first generation pan CDK inhibitor, targeting CDKs 1, 2, 4, 6, 7, and 9. It induces cell cycle arrest in ALL and AML cell lines as well as leukemia and lymphoma animal models [79,80]. Preclinical studies of flavopiridol in combination with BCL-2 inhibitor venetoclax or pan BH3 mimetic obatoclax revealed synergistic anti-apoptotic effects in vitro and in vivo, probably mediated via MCL-1, BIM, and NOXA regulation [81,82]. Flavopiridol has also been tested in CML and acted synergistically with pro-apoptotic pyrrolo-1,5-benzoxazepine compounds in imatinib-resistant cells. The observed induction of apoptosis was likely due to deactivation of the CDK1/cyclin B1 complex [83].

Clinical efficacy was observed in hematological neoplasms including CLL and AML, especially in AML as part of the FLAM regimen (flavopiridol followed by cytarabine and mitoxanthrone) [6,84]. Clinical investigation in CLL with early trials using flavopiridol achieved PR in almost half of the patients [85]. Further, a clinical trial is currently evaluating the potential of flavopiridol and decitabine in myelodysplastic syndrome. However, the use of flavopiridol proved difficult in the clinical setting, offering a complex pharmacokinetic profile, a wide range of side effects and an unclear mechanism of action [12].

Several mechanisms of resistance have been described for flavopiridol (Figure 2). Mahoney et al. identified endoplasmatic reticulum stress-mediated death of CLL cells as a novel mode of action for flavopiridol. However, induction of autophagy decreased cytotoxic effects while autophagy inhibition supported stress-mediated anti-leukemic effects, highlighting autophagy as a potential mechanism of flavopiridol resistance [86]. Besides, members of the BCL-2/MCL-1 apoptosis signaling cascade are involved in flavopiridol resistance. Data by Yeh et al. indicated that prolonged MCL-1 stability, in line with RNA polymerase II phosphorylation and CDK9 kinase domain upregulation, contributes to resistance in B-ALL cell line 697. MCL-1 upregulation was probably mediated via MAPK/ERK signaling. Knockdown of MCL-1 restored flavopiridol sensitivity and induced cytotoxicity [87]. Besides MCL-1, BCL-2 is another anti-apoptotic signaling molecule involved in flavopiridol metabolism. Decker et al. demonstrated that a truncated BCL-2 protein lacking the phosphorylation loop domain results in flavopiridol resistance in U937 AML cells [88].

### 4.2. Dinaciclib

Dinaciclib (SCH727965) is now widely evaluated in hematological malignancies. By inhibiting CDKs 1, 2, 5, and 9, dinaciclib reduces cell viability, induces apoptosis and cell cycle arrest in ALL and AML cell lines, also with *MLL* rearrangement, primary patient cells, and in vivo models [89,90,91,92]. A phase II study found reduced numbers of circulating blasts but no bone marrow remission in AML and ALL patients [91].

In CLL, dinaciclib downregulates MCL-1 gene and protein expression and potently induces apoptosis in patient-derived cells [93]. Interestingly, this effect was also present when the cells were cultured with cytokines produced by microenvironment cells but not with direct stromal cell contact. This lack of therapeutic efficacy was overcome by addition of PI3Kα inhibitor PIK-75 but not inhibitors of other PI3K isoforms [93]. Chen et al. subsequently characterized dinaciclib-induced effects on signaling pathways, elucidating caspase 8 and 9-mediated apoptosis induction with MCL-1 and BCL-XL suppression. They further detected inhibition of oncogenic pathways including STAT3, NFkB, p38, PI3K/AKT, and MAPK [94]. In addition, dinaciclib enhanced ibrutinib and venetoclax sensitivity. When combined with SYK inhibitor entospletinib, no synergistic effect was seen. Two phase I clinical trials evaluated the effect of dinaciclib in R/R CLL; combined with rituximab, four out of five patients had SD and one achieved CR [95]. In combination with ofatumumab, a median progression-free survival of 322 days was observed [96].

In mantle cell lymphoma, Höring et al. pursued a combined MCL-1 inhibiting and NOXA stabilizing approach using dinaciclib and fatty acid synthase inhibitor orlistat. They observed synergistically-induced NOXA-dependent apoptosis in mantle cell lymphoma cell lines and primary samples along with tumor growth inhibition in vivo [97]. In advanced non-Hodgkin lymphoma, B-CLL, and myeloma, no PR, CR, or SD was observed in a clinical phase I study with dinaciclib alone [98]. A different study reported SD in eight out of 61 advanced non-Hodgkin lymphoma or myeloma patients treated with dinaciclib in combination with aprepitant, ondansetron, and dexamethasone [99].

Further preclinical evaluation detected increased doxorubicin response rates in myeloma cell lines after low dose dinaciclib treatment, boosting growth inhibition and promoting senescence [100]. Interestingly, and in contrast to several other studies of dinaciclib in hematological neoplasms, this work did not observe apoptosis induction but accelerated senescence via increased p16 signaling. Additionally, dinaciclib may have reduced doxorubicin-induced ATM/Chk2/p53/p21 senescence-modulating signaling. Further investigation in myeloma was conducted by Alagpulinsa et al. who combined dinaciclib with PARP inhibitor ABT-888, based on the idea of impaired homologous recombination during PARP-mediated DNA double strand break repair after dinaciclib [101]. Dinaciclib-treated myeloma cells had increased DNA damage and reduced repair gene expression. Cotreatment with PARP inhibitor ABT-888 synergistically reduced tumor cell proliferation in vitro as well as in vivo using myeloma xenograft models. A subsequent phase I/II study found an overall response rate of 11% in myeloma patients receiving dinaciclib alone [102]. A combinatory approach with bortezomib is currently being evaluated in a phase I plasma cell myeloma study.

### 4.3. Other Pan CDK Inhibitors Investigated in Clinical Trials

Further pan CDK inhibitors are now also tested in the clinical setting. Besides the CDKs targeted by dinaciclib (CDK1, 2, 5, 9), AT7519M also inhibits CDK4, still inducing apoptosis in vitro and in vivo and reducing proliferation in myeloma and CLL [103,104]. Two clinical trials investigating AT7519M in non-Hodgkin lymphoma as well as R/R CLL and mantle cell lymphoma observed SD in at least half of the patients enrolled and PR in three out of 12 R/R mantle cell lymphoma cases [105,106].

Voruciclib (P1446A), a CDK1/2/4/5/6/8/9 inhibitor, was evaluated in a phase I study recruiting follicular lymphoma, mantle cell lymphoma, marginal zone lymphoma, small lymphocytic lymphoma, CLL, diffuse large B-cell lymphoma, and AML patients after demonstrating significant potential in preclinical studies [61,107]. Combined application of voruciclib and BCL-2 inhibitor venetoclax was synergistic in preclinical AML models. Notably, venetoclax-mediated *MCL-1* and *c-Myc* downregulation was only reached in an intermittent drug administration schedule that should be considered in clinical practice [108].

The CDK1/4/9 inhibitor P276-00 showed promising in vitro and in vivo results in AML [109], myeloma [110], and mantle cell lymphoma [111] but only achieved SD in two out of 13 R/R mantle cell lymphoma patients in a phase II clinical trial [112].

Roniciclib (BAY1000394) targets the same CDKs as flavopiridol (1, 2, 4, 6, 7, and 9) and achieved SD in three out of seven patients with lymphoid neoplasms [113]. Of note, this is the only clinical trial so far investigating CDKIs in classical Hodgkin lymphoma. Earlier studies suggested that there is a relation between apoptosis-induction, DNA fragmentation, survival, and expression of cell cycle regulators p27 and p21 [114], providing a rationale for CDK targeting in Hodgkin lymphoma. Sánchez-Aguilera et al. further identified CDK regulator p18*^INK4C^* as a potential tumor suppressor gene in Hodgkin lymphoma [115]. Finally, loss of p16*^INK4A^* expression has been observed in Reed–Sternberg cells of Hodgkin lymphoma cases [116].

Due to its variety in mechanistic pathways, pan CDK inhibition offers a broad range of possibilities to target virtually every hematological subtype. Most progress has been made for dinaciclib, which is already investigated in clinical trials; however, results are ambiguous. Chemical modifications or combinatorial schedules might be beneficial to increase the therapeutic outcome.

## 5. Dual Kinase Inhibitors and Novel Approaches

Developing resistance to CDKI monotherapy is frequently seen in both preclinical and clinical studies. Hence, novel approaches are being evaluated, including dual kinase inhibitors. In the AML setting, most inhibitors target one or more CDKs and FLT3. AMG925 and FN-1501 inhibit FLT3 in combination with CDK4 and CDK2/4/6, respectively. Both drugs significantly induced apoptosis and anti-leukemic effects as well as ERK/AKT/Rb dephosphorylation in vitro and in vivo [117,118].

TG02, a pan CDK and FLT3 inhibitor, further targets JAK2. This inhibition promoted G1 arrest, apoptosis, and tumor regression in AML cell lines, in vivo models, and primary samples [119,120,121]. TG02 is also effective in myeloma cell lines as single agent under protective bone marrow niche conditions and in xenografts. TG02 further demonstrated synergistic potential with approved anti-myeloma agents dexamethasone, melphalan, bortezomib, and lenalidomide, possibly via ERK5 blockade, intrinsic and extrinsic apoptosis induction, and cell cycle blockade [122].

Additionally in myeloma, pan CDK/JAK/Src/AMPK/GSK3β inhibitor RGB-286638 achieved cytotoxicity in vitro and prolonged animal survival [123]. Besides CDK4 and CDK6, ON123300 and ON108110 inhibit PI3Kδ and CK2, respectively, and induced Rb dephosphorylation, G1 arrest, and apoptosis in mantle cell lymphoma [124,125].

Structural similarity of CDK ATP binding sites is a major challenge in the design and development of selective inhibitors. Proteolysis-targeting chimeras (PROTACs) are a novel approach of CDK elimination. These synthetic molecules comprise a ligand for the protein of interest, for example, CDK6, and another ligand which recruits E3 ligases. E3 ligases then induce ubiquitination and proteasomal degradation of the target structure. In a recent study of De Dominici et al. investigating BCR-ABL1-positive ALL, PROTAC YX-2-107 was designed to bind CDK4/6, reducing CDK6 enzymatic activity and inducing degradation in vitro. Further, S phase transition was suppressed and Rb and FOXM1 dephosphorylation was observed. In vivo, PROTAC YX-2-107 achieved a comparable or better reduced leukemia burden in PDX mice compared to palbociclib [126]. Another PROTAC, ARV-771, targets BET and acts synergistically with palbociclib in ibrutinib-resistant mantle cell lymphoma cells [127]. PROTACs have also been designed to inhibit other CDKs. Qiu et al. designed a series of PROTACs based on CDK9 inhibitor atuveciclib (BAY-1143572) and showed anti-leukemic effects at low nanomolar concentrations, also in vivo [128].

Novel and innovative strategies like dual kinase inhibition or PROTAC design are important steps towards an improved targeted therapy. Further preclinical studies in syngeneic and xenograft models are needed to identify candidates with a high likelihood of proving beneficial in a clinical setting.

## 6. The Interaction of CDKIs with the Tumor Microenvironment

A major challenge for receiving long-term disease free-survival in hematological diseases is successful targeting of the malignant niche in the bone marrow. The latter is a complex microenvironment in which hematopoietic stem cells interact with multiple non-hematopoietic cell types. Just like normal stem cells, leukemia stem cells, hosted in the stem cell niche, undergo self-renewal, can efflux drugs, and have a quiescent cell cycle status, which makes them difficult to target. Especially CDK6 plays a critical role in hematopoietic stem cell differentiation [129]. A comparable role in leukemia stem cells can be expected.

Ischemia-like conditions are the driving force of leukemia stem cell refractoriness to classical drugs as well as targeted agents, such as imatinib mesylate [130]. Though experimental evidence for the interaction of CDKIs with hypoxic niches of the bone marrow is still pending, experiences from solid tumor models described successful reversal of hypoxia-mediated therapy resistance via CDK1 and CDK2 interaction with hypoxia inducible factor-1 [131]. The mechanism was due to Rb hypophosphorylation by palbociclib, which favors cell cycle arrest and senescence. A recent case report identified rebound lymphocytosis in a CLL patient after terminating palbociclib treatment for synchronic breast cancer [132]. This report impressively demonstrates the interaction of CDK4/6 inhibitors with the tumor microenvironment via induction of a cell cycle arrest. Still, follow-up studies should focus on the stem cell niche to judge the efficacy of CDKIs and ultimately prevent relapse.

## 7. Conclusions

The field of hematological neoplasms is very heterogeneous with each entity featuring individual characteristics and challenges. However, most of them are still difficult to treat, indicated by high relapse rates, intimidating prognoses, and lack of durable curative therapies. CDKs and their regulating cyclins are of major importance for the tumorigenic potential of developing leukemia or lymphoma cells and frequently dysregulated in those malignancies. Modulation of CDK expression and activity thus represents a promising strategy to target aberrant cell cycle progression and proliferation in hematological tumor cells.

The results of extensive preclinical evaluations are promising, with several clinical studies currently testing CDKIs in most hematological malignancies. CDK4/6Is palbociclib, abemaciclib, and ribociclib are already FDA-approved for solid neoplasms, raising hopes for a subsequent clinical implementation in hematological malignancies in the near future. Interestingly, clinical trials have mainly been finished for lymphoma (especially mantle cell lymphoma) and CLL patients while data on acute leukemias are lagging behind. Published results indicate that combined therapy with other small molecule inhibitors like ibrutinib or bortezomib as well as inhibitors of anti-apoptotic proteins may have the capacity to circumvent CDKI resistance development. Importantly, most clinical studies so far have been conducted in heavily pretreated relapsed/refractory patient cohorts, highlighting the potential of CDK targeting for hematological malignancy management. 

Another open question that must be addressed in future studies is why inhibitors of the same class result in highly variable response rates in the same entity. This underlines the importance of further investigation on biomarkers, resistance mechanisms, and exact modes of action.

This is an exciting period potentially improving the therapeutic outcome of leukemia and lymphoma patients; however, there are still unanswered questions and several issues to be addressed. Clinical data on acute leukemias are still missing while results in other entities are ambiguous or obtained from rather small cohorts. Appropriate combination partners need to be identified, ideal application sequences and dosages are to be evaluated, and biomarkers are necessary to offer a useful and potent CDKI-based therapy for the special needs of every molecular subgroup in a variety of hematological entities.

## Supplementary Materials

The following are available online at https://www.mdpi.com/article/10.3390/cancers13102497/s1, Table S1: Overview on recent preclinical and clinical application of CDK inhibitors in hematological malignancies as well as potential combination partners showing boosted effects.

## Abbreviations

*ABL1*: ABL proto-oncogene 1, non-receptor tyrosine kinase; ADP, adenosine diphosphate; AFF, AF4/FMR2 gene family; AKT, AKT serine/threonine kinase; ALL, acute lymphoblastic leukemia; AML, acute myeloid leukemia; AMPK, protein kinase AMP; Apaf1, apoptotic peptidase activating factor 1; ATM, ATM serine/threonine kinase; ATP, adenosine triphosphate; AURORA, Aurora kinase; BAK, BCL2 antagonist/killer 1; BAX, BCL2 associated X, apoptosis regulator; BCL-2, *BCL2* gene, BCL2 apoptosis regulator; BCL-XL, *BCL2L1* gene, BCL2 like 1 *BCR*, BCR activator of RhoGEF and GTPase; BET, bromodomain and extra-terminal motif proteins; BH3, BH3 domain; BIM, *BCL2L11* gene, BCL2 like 11; c-MYC, *MYC* proto-oncogene, BHLH transcription factor; Casp, Caspase; *CCND1*, cyclin D1; *CCND2*, cyclin D2; CD25, *IL2RA* gene, interleukin 2 receptor subunit alpha; CD40L, CD40 ligand; CDK, cyclin dependent kinase; CDKI, cyclin dependent kinase inhibitor; Chk2, checkpoint kinase 2; CK2, casein kinase 2; CLL, chronic lymphoblastic leukemia; CML, chronic myeloid leukemia; CR, complete remission; DLBCL, diffuse large B-cell lymphoma; DNA, deoxy ribonucleic acid; E2F, E2F transcription factor gene family; ERK, mitogen-activated protein kinase family pathway; *ETO*, *RUNX1T1* gene, RUNX1 partner transcriptional co-repressor 1; EZH2, Enhancer of zeste 2 polycomb repressive complex 2 subunit; FAS, Fas cell surface death receptor; FAS-L, Fas ligand; FDA, U.S. Food and Drug Administration; *FGFR1*, fibroblast growth factor receptor 1; FLAM, fludarabine, cytarabine and mitoxanthrone; *FLT3*, Fms related receptor tyrosine kinase 3; *FOXO3*, forkhead box O3; *FOXM1*, forkhead box M1; GTP, guanosine triphosphate; GSK3, glycogen synthase kinase 3; HDAC, histone deacetylase; *HOX*, homeobox gene family; ITD, internal tandem duplication mutation in the *FLT3* gene; JAK2, janus kinase 2; MAPK, mitogen-activated protein kinase family pathway; MCL, mantle cell lymphoma; *MCL1*, MCL1 apoptosis regulator, BCL2 family member; *MLL*, *KMT2A* gene, lysine methyltransferase 2A; MM, multiple myeloma; mTOR, mechanistic target of rapamycin kinase; NFkB, nuclear factor kappa B *NOTCH1*, Notch receptor 1 gene; NOXA, PMAIP1 gene, phorbol-12-myristate-13-acetate-induced protein 1; p16*^INK4A^*, *CDKN2A* gene, cyclin dependent kinase inhibitor 2A; p18*^INK4C^*, *CDKN2C* gene, cyclin dependent kinase inhibitor 2C; p21, *CDKN1A* gene, cyclin dependent kinase inhibitor 1A; p27, *CDKN1B* gene, cyclin dependent kinase inhibitor 1B; p38, *MAPK14* gene, mitogen-activated protein kinase 14; p53, *TP53* gene, tumor protein 53; PARP, Poly(ADP-ribose) polymerase family; PBOX, pro-apoptotic compounds; PDX, patient derived xenograft; PI3K, phosphatidyl inositol 3 kinase; PR, partial response; PROTAC, proteolysis targeting chimera; Ras, Ras proto-oncogene, GTPase family; Rb, RB transcriptional corepressor 1; RNA, ribonucleic acid; ROS, reactive oxygen species; *RUNX1*, RUNX family transcription factor 1; R/R, relapsed/refractory; SD, stable disease; Src, *SRC* proto-oncogene, non-receptor tyrosine kinase; STAT3, signal transducer and activator of transcription 3; SYK, spleen associated tyrosine kinase; TKD, tyrosine kinase domain mutation in the *FLT3* gene; XIAP, X-linked inhibitor of apoptosis.

## References

[1] Hunger S.P., Mullighan C.G. (2015). Redefining ALL classification: Toward detecting high-risk ALL and implementing precision medicine. Blood.

[2] Abdul Hamid G., Hariri F. (2019). Introductory Chapter: Advances in Hematologic Malignancies. Advances in Hematologic Malignancies.

[3] Krok-Schoen J.L., Fisher J.L., Stephens J.A., Mims A., Ayyappan S., Woyach J.A., Rosko A.E. (2018). Incidence and survival of hematological cancers among adults ages ≥75 years. Cancer Med..

[4] Pulte D., Jansen L., Brenner H. (2016). Most up-to-Date Long Term Survival Estimates for Common Hematologic Malignancies Using the Boomerang Method. Blood.

[5] Lee D.J., Zeidner J.F. (2019). Cyclin-dependent kinase (CDK) 9 and 4/6 inhibitors in acute myeloid leukemia (AML): A promising therapeutic approach. Expert Opin. Investig. Drugs.

[6] Aleem E., Arceci R.J. (2015). Targeting cell cycle regulators in hematologic malignancies. Front. Cell Dev. Biol..

[7] Bradford G.B., Williams B., Rossi R., Bertoncello I. (1997). Quiescence, cycling, and turnover in the primitive hematopoietic stem cell compartment. Exp. Hematol..

[8] Cheshier S.H., Morrison S.J., Liao X., Weissman I.L. (1999). In vivo proliferation and cell cycle kinetics of long-term self-renewing hematopoietic stem cells. Proc. Natl. Acad. Sci. USA.

[9] Orford K.W., Scadden D.T. (2008). Deconstructing stem cell self-renewal: Genetic insights into cell-cycle regulation. Nat. Rev. Genet..

[10] Weissman I.L. (2000). Stem cells: Units of development, units of regeneration, and units in evolution. Cell.

[11] Grant S., Dent P. (2007). Simultaneous Interruption of Signal Transduction and Cell Cycle Regulatory Pathways: Implications for New Approaches to the Treatment of Childhood Leukemias. Curr. Drug Targets.

[12] Dickson M.A. (2014). Molecular pathways: CDK4 inhibitors for cancer therapy. Clin. Cancer Res..

[13] Antony-Debré I., Steidl U. (2014). CDK6, a new target in MLL-driven leukemia. Blood.

[14] Van Der Linden M.H., Willekes M., Roon E., Seslija L., Schneider P., Pieters R., Stam R.W. (2014). MLL fusion-driven activation of CDK6 potentiates proliferation in MLL-rearranged infant ALL. Cell Cycle.

[15] Placke T., Faber K., Nonami A., Putwain S.L., Salih H.R., Heidel F.H., Krämer A., Root D.E., Barbie D.A., Krivtsov A.V. (2014). Requirement for CDK6 in MLL-rearranged acute myeloid leukemia. Blood.

[16] Krivtsov A.V., Feng Z., Lemieux M.E., Faber J., Vempati S., Sinha A.U., Xia X., Jesneck J., Bracken A.P., Silverman L.B. (2008). H3K79 methylation profiles define murine and human MLL-AF4 leukemias. Cancer Cell.

[17] Wang L., Wang J., Blaser B.W., Duchemin A.M., Kusewitt D.F., Liu T., Caligiuri M.A., Briesewitz R. (2007). Pharmacologic inhibition of CDK4/6: Mechanistic evidence for selective activity or acquired resistance in acute myeloid leukemia. Blood.

[18] Parylo S., Vennepureddy A., Dhar V., Patibandla P., Sokoloff A. (2019). Role of cyclin-dependent kinase 4/6 inhibitors in the current and future eras of cancer treatment. J. Oncol. Pharm. Pract..

[19] Kathiravan M., Singh M., Bhatia P., Trehan A., Varma N., Sachdeva M.S., Bansal D., Jain R., Naseem S. (2019). Deletion of CDKN2A/B is associated with inferior relapse free survival in pediatric B cell acute lymphoblastic leukemia. Leuk. Lymphoma.

[20] Uras I.Z., Walter G.J., Scheicher R., Bellutti F., Prchal-Murphy M., Tigan A.S., Valent P., Heidel F.H., Kubicek S., Scholl C. (2016). Palbociclib treatment of FLT3-ITD+ AML cells uncovers a kinase-dependent transcriptional regulation of FLT3 and PIM1 by CDK6. Blood.

[21] Uras I.Z., Maurer B., Nebenfuehr S., Zojer M., Valent P., Sexl V. (2018). Therapeutic vulnerabilities in FLT3-mutant aml unmasked by palbociclib. Int. J. Mol. Sci..

[22] Martinez-Soria N., McKenzie L., Draper J., Ptasinska A., Issa H., Potluri S., Blair H.J., Pickin A., Isa A., Chin P.S. (2018). The Oncogenic Transcription Factor RUNX1/ETO Corrupts Cell Cycle Regulation to Drive Leukemic Transformation. Cancer Cell.

[23] Matsuo H., Yoshida K., Fukumura K., Nakatani K., Noguchi Y., Takasaki S., Noura M., Shiozawa Y., Shiraishi Y., Chiba K. (2018). Recurrent CCND3 mutations in MLL-rearranged acute myeloid leukemia. Blood Adv..

[24] Chen X., Burkhardt D.B., Hartman A.A., Hu X., Eastman A.E., Sun C., Wang X., Zhong M., Krishnaswamy S., Guo S. (2019). MLL-AF9 initiates transformation from fast-proliferating myeloid progenitors. Nat. Commun..

[25] Agirre X., Vilas-Zornoza A., Jiménez-Velasco A., Martin-Subero J.I., Cordeu L., Gárate L., José-Eneriz E.S., Abizanda G., Rodríguez-Otero P., Fortes P. (2009). Epigenetic silencing of the tumor suppressor microRNA Hsa-miR-124a regulates CDK6 expression and confers a poor prognosis in acute lymphoblastic leukemia. Cancer Res..

[26] Sawai C.M., Freund J., Oh P., Ndiaye-Lobry D., Bretz J.C., Strikoudis A., Genesca L., Trimarchi T., Kelliher M.A., Clark M. (2012). Therapeutic Targeting of the Cyclin D3:CDK4/6 Complex in T Cell Leukemia. Cancer Cell.

[27] Choi Y.J., Li X., Hydbring P., Sanda T., Stefano J., Christie A.L., Signoretti S., Look A.T., Kung A.L., von Boehmer H. (2012). The Requirement for Cyclin D Function in Tumor Maintenance. Cancer Cell.

[28] Nemoto A., Saida S., Kato I., Kikuchi J., Furukawa Y., Maeda Y., Akahane K., Honna-Oshiro H., Goi K., Kagami K. (2016). Specific antileukemic activity of PD0332991, a CDK4/6 inhibitor, against philadelphia chromosome-positive lymphoid leukemia. Mol. Cancer Ther..

[29] Baughn L.B., Di Liberto M., Wu K., Toogood P.L., Louie T., Gottschalk R., Niesvizky R., Cho H., Ely S., Moore M.A.S. (2006). A novel orally active small molecule potently induces G1 arrest in primary myeloma cells and prevents tumor growth by specific inhibition of cyclin-dependent kinase 4/6. Cancer Res..

[30] Menu E., Garcia J., Huang X., Di Liberto M., Toogood P.L., Chen I., Vanderkerken K., Chen-Kiang S. (2008). A novel therapeutic combination using PD 0332991 and bortezomib: Study in the 5T33MM myeloma model. Cancer Res..

[31] Altenburg J.D., Farag S.S. (2015). The potential role of PD0332991 (Palbociclib) in the treatment of multiple myeloma. Expert Opin. Investig. Drugs.

[32] Chaturvedi N.K., Hatch N.D., Sutton G.L., Kling M., Vose J.M., Joshi S.S. (2019). A novel approach to eliminate therapy-resistant mantle cell lymphoma: Synergistic effects of Vorinostat with Palbociclib. Leuk. Lymphoma.

[33] Chiron D., Di Liberto M., Martin P., Huang X., Sharman J., Blecua P., Mathew S., Vijay P., Eng K., Ali S. (2014). Cell-cycle reprogramming for Pi3K inhibition overrides a relapse-specific C481s BTK mutation revealed by longitudinal functional genomics in mantle cell lymphoma. Cancer Discov..

[34] Hoareau-Aveilla C., Quelen C., Congras A., Caillet N., Labourdette D., Dozier C., Brousset P., Lamant L., Meggetto F. (2019). miR-497 suppresses cycle progression through an axis involving CDK6 in ALK-positive cells. Haematologica.

[35] Leonard J.P., LaCasce A.S., Smith M.R., Noy A., Chirieac L.R., Rodig S.J., Yu J.Q., Vallabhajosula S., Schoder H., English P. (2012). Selective CDK4/6 inhibition with tumor responses by PD0332991 in patients with mantle cell lymphoma. Blood.

[36] A Study Of Oral Palbociclib (PD-0332991), A Cyclin-Dependent Kinase Inhibitor, In Patients With Advanced Cancer—Study Results—ClinicalTrials.gov. https://clinicaltrials.gov/ct2/show/results/NCT00141297?term=NCT00141297&draw=2&rank=1.

[37] Niesvizky R., Badros A.Z., Costa L.J., Ely S.A., Singhal S.B., Stadtmauer E.A., Haideri N.A., Yacoub A., Hess G., Lentzsch S. (2015). Phase 1/2 study of cyclin-dependent kinase (CDK)4/6 inhibitor palbociclib (PD-0332991) with bortezomib and dexamethasone in relapsed/refractory multiple myeloma. Leuk. Lymphoma.

[38] Martin P., Ruan J., Furman R., Rutherford S., Allan J., Chen Z., Huang X., DiLiberto M., Chen-Kiang S., Leonard J.P. (2019). A phase I trial of palbociclib plus bortezomib in previously treated mantle cell lymphoma. Leuk. Lymphoma.

[39] Martin P., Bartlett N.L., Blum K.A., Park S., Maddocks K., Ruan J., Ridling L.A., Dittus C., Chen Z., Huang X. (2019). A phase 1 trial of ibrutinib plus palbociclib in previously treated mantle cell lymphoma. Blood.

[40] Yang C., Boyson C.A., Di Liberto M., Huang X., Hannah J., Dorn D.C., Moore M.A.S., Chen-Kiang S., Zhou P. (2015). CDK4/6 inhibitor PD 0332991 sensitizes acute myeloid leukemia to cytarabine-mediated cytotoxicity. Cancer Res..

[41] Rangatia J., Bonnet D. (2006). Transient or long-term silencing of BCR-ABL alone induces cell cycle and proliferation arrest, apoptosis and differentiation. Leukemia.

[42] Schneeweiss-Gleixner M., Byrgazov K., Stefanzl G., Berger D., Eisenwort G., Lucini C.B., Herndlhofer S., Preuner S., Obrova K., Pusic P. (2019). CDK4/CDK6 inhibition as a novel strategy to suppress the growth and survival of BCR-ABL1T315I+ clones in TKI-resistant CML. EBioMedicine.

[43] Rodriguez-Otero P., Román-Gómez J., Vilas-Zornoza A., José-Eneriz E.S., Martín-Palanco V., Rifón J., Torres A., Calasanz M.J., Agirre X., Prosper F. (2011). Deregulation of FGFR1 and CDK6 oncogenic pathways in acute lymphoblastic leukaemia harbouring epigenetic modifications of the MIR9 family. Br. J. Haematol..

[44] Kuo T.C., Chavarria-Smith J.E., Huang D., Schlissel M.S. (2011). Forced Expression of Cyclin-Dependent Kinase 6 Confers Resistance of Pro-B Acute Lymphocytic Leukemia to Gleevec Treatment. Mol. Cell. Biol..

[45] Chiron D., Martin P., Di Liberto M., Huang X., Ely S., Lannutti B.J., Leonard J.P., Mason C.E., Chen-Kiang S. (2013). Induction of prolonged early G1 arrest by CDK4/CDK6 inhibition reprograms lymphoma cells for durable PI3Kd inhibition through PIK3IP1. Cell Cycle.

[46] Sun B., Shah B., Fiskus W., Qi J., Rajapakshe K., Coarfa C., Li L., Devaraj S.G.T., Sharma S., Zhang L. (2015). Synergistic activity of BET protein antagonist-based combinations in mantle cell lymphoma cells sensitive or resistant to ibrutinib. Blood.

[47] Kozaki R., Vogler M., Walter H.S., Jayne S., Dinsdale D., Siebert R., Dyer M.J.S., Yoshizawa T. (2018). Responses to the selective bruton’s tyrosine kinase (BTK) inhibitor tirabrutinib (ONO/GS-4059) in diffuse large B-cell lymphoma cell lines. Cancers.

[48] Tao Y.F., Wang N.N., Xu L.X., Li Z.H., Li X.L., Xu Y.Y., Fang F., Li M., Qian G.H., Li Y.H. (2017). Molecular mechanism of G1 arrest and cellular senescence induced by LEE011, a novel CDK4/CDK6 inhibitor, in leukemia cells. Cancer Cell Int..

[49] Bortolozzi R., Mattiuzzo E., Trentin L., Accordi B., Basso G., Viola G. (2018). Ribociclib, a Cdk4/Cdk6 kinase inhibitor, enhances glucocorticoid sensitivity in B-acute lymphoblastic leukemia (B-All). Biochem. Pharmacol..

[50] Pikman Y., Alexe G., Roti G., Conway A.S., Furman A., Lee E.S., Place A.E., Kim S., Saran C., Modiste R. (2017). Synergistic drug combinations with a CDK4/6 inhibitor in T-cell acute lymphoblastic leukemia. Clin. Cancer Res..

[51] Jena N., Sheng J., Hu J.K., Li W., Zhou W., Lee G., Tsichlis N., Pathak A., Brown N., Deshpande A. (2016). CDK6-mediated repression of CD25 is required for induction and maintenance of Notch1-induced T-cell acute lymphoblastic leukemia. Leukemia.

[52] Infante J.R., Shapiro G.I., Witteveen P.O., Gerecitano J.F., Ribrag V., Chugh R., Chakraborty A., Matano A., Zhao X., Parasuraman S. (2013). Abstract A276: Phase 1 multicenter, open label, dose-escalation study of LEE011, an oral inhibitor of cyclin-dependent kinase 4/6, in patients with advanced solid tumors or lymphomas. Mol. Cancer Ther. Am. Assoc. Cancer Res..

[53] Tanaka Y., Momose S., Tabayashi T., Sawada K., Yamashita T., Higashi M., Sagawa M., Tokuhira M., Rosenwald A., Kizaki M. (2020). Abemaciclib, a CDK4/6 inhibitor, exerts preclinical activity against aggressive germinal center-derived B-cell lymphomas. Cancer Sci..

[54] Study of LY2835219 for Mantle Cell Lymphoma—Study Results—ClinicalTrials.gov. https://clinicaltrials.gov/ct2/show/results/NCT01739309?term=NCT01739309&draw=2&rank=1.

[55] Morschhauser F., Bouabdallah K., Stilgenbauer S., Thieblemont C., de Guibert S., Zettl F., Gelbert L.M., Turner P.K., Kambhampati S.R.P., Li L. (2020). Clinical activity of abemaciclib in patients with relapsed or refractory mantle cell lymphoma —A phase II study. Haematologica.

[56] Gelbert L.M., Cai S., Lin X., Sanchez-Martinez C., Prado M.D., Lallena M.J., Torres R., Ajamie R.T., Wishart G.N., Flack R.S. (2014). Preclinical characterization of the CDK4/6 inhibitor LY2835219: In-vivo cell cycle-dependent/independent anti-tumor activities alone/in combination with gemcitabine. Invest. N. Drugs.

[57] Iriyama N., Hino H., Moriya S., Hiramoto M., Hatta Y., Takei M., Miyazawa K. (2018). The cyclin-dependent kinase 4/6 inhibitor, abemaciclib, exerts dose-dependent cytostatic and cytocidal effects and induces autophagy in multiple myeloma cells. Leuk. Lymphoma.

[58] Nakatani K., Matsuo H., Harata Y., Higashitani M., Koyama A., Noura M., Nishinaka-Arai Y., Kamikubo Y., Adachi S. (2020). Inhibition of CDK4/6 and autophagy synergistically induces apoptosis in t(8;21) acute myeloid leukemia cells. Int. J. Hematol..

[59] Bisi J.E., Sorrentino J.A., Jordan J.L., Darr D.D., Roberts P.J., Tavares F.X., Strum J.C. (2017). Preclinical development of G1T38: A novel, potent and selective inhibitor of cyclin dependent kinases 4/6 for use as an oral antineoplastic in patients with CDK4/6 sensitive tumors. Oncotarget.

[60] Bacon C.W., D’Orso I. (2019). CDK9: A signaling hub for transcriptional control. Transcription.

[61] Dey J., Deckwerth T.L., Kerwin W.S., Casalini J.R., Merrell A.J., Grenley M.O., Burns C., Ditzler S.H., Dixon C.P., Beirne E. (2017). Voruciclib, a clinical stage oral CDK9 inhibitor, represses MCL-1 and sensitizes high-risk Diffuse Large B-cell Lymphoma to BCL2 inhibition. Sci. Rep..

[62] Cidado J., Boiko S., Proia T., Ferguson D., Criscione S.W., Martin M.S., Pop-Damkov P., Su N., Franklin V.N.R., Chilamakuri C.S.R. (2020). AZD4573 is a highly selective CDK9 inhibitor that suppresses Mcl-1 and induces apoptosis in hematologic cancer cells. Clin. Cancer Res..

[63] Walsby E., Pratt G., Shao H., Abbas A.Y., Fischer P.M., Bradshaw T.D., Brennan P., Fegan C., Wang S., Pepper C. (2014). A novel Cdk9 inhibitor preferentially targets tumor cells and synergizes with fludarabine. Oncotarget.

[64] Xie S., Wei F., Sun Y., Gao Y., Pan L., Tan M., Wang S., Ding J., Chen Y. (2019). EZH2 inhibitors abrogate upregulation of trimethylation of H3K27 by CDK9 inhibitors and potentiate its activity against diffuse large B-cell lymphoma. Haematologica.

[65] Xie S., Jiang H., Zhai X.W., Wei F., Wang S.D., Ding J., Chen Y. (2016). Antitumor action of CDK inhibitor LS-007 as a single agent and in combination with ABT-199 against human acute leukemia cells. Acta Pharmacol. Sin..

[66] Chukkapalli V., Gordon L.I., Venugopal P., Borgia J.A., Karmali R. (2018). Metabolic changes associated with metformin potentiates Bcl-2 inhibitor, Venetoclax, and CDK9 inhibitor, BAY1143572 and reduces viability of lymphoma cells. Oncotarget.

[67] Lücking U., Scholz A., Lienau P., Siemeister G., Kosemund D., Bohlmann R., Briem H., Terebesi I., Meyer K., Prelle K. (2017). Identification of Atuveciclib (BAY 1143572), the First Highly Selective, Clinical PTEFb/CDK9 Inhibitor for the Treatment of Cancer. ChemMedChem.

[68] Göthert J.R., Imsak R., Möllmann M., Kesper S., Göbel M., Dührsen U., Scholz A., Lücking U., Baumann M., Unger A. (2018). Potent anti-leukemic activity of a specific cyclin-dependent kinase 9 inhibitor in mouse models of chronic lymphocytic leukemia. Oncotarget.

[69] Bharate S.B., Kumar V., Jain S.K., Mintoo M.J., Guru S.K., Nuthakki V.K., Sharma M., Bharate S.S., Gandhi S.G., Mondhe D.M. (2018). Discovery and Preclinical Development of IIIM-290, an Orally Active Potent Cyclin-Dependent Kinase Inhibitor. J. Med. Chem..

[70] Phillips D.C., Jin S., Gregory G.P., Zhang Q., Xue J., Zhao X., Chen J., Tong Y., Zhang H., Smith M. (2019). A novel CDK9 inhibitor increases the efficacy of venetoclax (ABT-199) in multiple models of hematologic malignancies. Leukemia.

[71] Wang B., Wu J., Wu Y., Chen C., Zou F., Wang A., Wu H., Hu Z., Jiang Z., Liu Q. (2018). Discovery of 4-(((4-(5-chloro-2-(((1s,4s)-4-((2-methoxyethyl)amino)cyclohexyl)amino)pyridin-4-yl)thiazol-2-yl)amino)methyl)tetrahydro-2H-pyran-4-carbonitrile (JSH-150) as a novel highly selective and potent CDK9 kinase inhibitor. Eur. J. Med. Chem..

[72] Poss Z.C., Ebmeier C.C., Taatjes D.J. (2013). The Mediator complex and transcription regulation. Crit. Rev. Biochem. Mol. Biol..

[73] Menzl I., Zhang T., Berger-Becvar A., Grausenburger R., Heller G., Prchal-Murphy M., Edlinger L., Knab V.M., Uras I.Z., Grundschober E. (2019). A kinase-independent role for CDK8 in BCR-ABL1+ leukemia. Nat. Commun..

[74] Rzymski T., Mikula M., Zylkiewicz E., Dreas A., Wiklik K., Golas A., Wójcik K., Masiejczyk M., Wróbel A., Dolata I. (2017). SEL120-34A is a novel CDK8 inhibitor active in AML cells with high levels of serine phosphorylation of STAT1 and STAT5 transactivation domains. Oncotarget.

[75] Sampathi S., Acharya P., Zhao Y., Wang J., Stengel K.R., Liu Q., Savona M.R., Hiebert S.W. (2019). The CDK7 inhibitor THZ1 alters RNA polymerase dynamics at the 5′ and 3′ ends of genes. Nucleic Acids Res..

[76] Zhang Y., Zhou L., Bandyopadhyay D., Sharma K., Allen A.J., Kmieciak M., Grant S. (2019). The covalent CDK7 inhibitor THz1 potently induces apoptosis in multiple myeloma cells in vitro and in vivo. Clin. Cancer Res..

[77] Hu S., Marineau J.J., Rajagopal N., Hamman K.B., Choi Y.J., Schmidt D.R., Ke N., Johannessen L., Bradley M.J., Orlando D.A. (2019). Discovery and characterization of SY-1365, a selective, covalent inhibitor of CDK7. Cancer Res..

[78] Park S.Y., Kim K.Y., Jun D.Y., Hwang S.K., Kim Y.H. (2020). G1 cell cycle arrest and extrinsic apoptotic mechanisms underlying the anti-leukemic activity of cdk7 inhibitor bs-181. Cancers.

[79] Parker B.W., Kaur G., Nieves-Neira W., Taimi M., Kohlhagen G., Shimizu T., Losiewicz M.D., Pommier Y., Sausville E.A., Senderowicz A.M. (1998). Early induction of apoptosis in hematopoietic cell lines after exposure to flavopiridol. Blood.

[80] Arguello F., Alexander M., Sterry J.A., Tudor G., Smith E.M., Kalavar N.T., Greene J.F., Koss W., Morgan C.D., Stinson S.F. (1998). Flavopiridol induces apoptosis of normal lymphoid cells, causes immunosuppression, and has potent antitumor activity In vivo against human leukemia and lymphoma xenografts. Blood.

[81] Bogenberger J., Whatcott C., Hansen N., Delman D., Shi C.X., Kim W., Haws H., Soh K., Lee Y.S., Peterson P. (2017). Combined venetoclax and alvocidib in acute myeloid leukemia. Oncotarget.

[82] Chen S., Dai Y., Pei X.Y., Myers J., Wang L., Kramer L.B., Garnett M., Schwartz D.M., Su F., Simmons G.L. (2012). CDK inhibitors upregulate BH3-only proteins to sensitize human myeloma cells to BH3 mimetic therapies. Cancer Res..

[83] Bright S.A., Campiani G., Deininger M.W., Lawler M., Williams D.C., Zisterer D.M. (2010). Sequential treatment with flavopiridol synergistically enhances pyrrolo-1,5-benzoxazepine-induced apoptosis in human chronic myeloid leukaemia cells including those resistant to imatinib treatment. Biochem. Pharmacol..

[84] Shafer D., Grant S. (2016). Update on rational targeted therapy in AML. Blood Rev..

[85] Byrd J.C., Lin T.S., Dalton J.T., Wu D., Phelps M.A., Fischer B., Moran M., Blum K.A., Rovin B., Brooker-McEldowney M. (2007). Flavopiridol administered using a pharmacologically derived schedule is associated with marked clinical efficacy in refractory, genetically high-risk chronic lymphocytic leukemia. Blood.

[86] Mahoney E., Lucas D.M., Gupta S.V., Wagner A.J., Herman S.E.M., Smith L.L., Yeh Y.Y., Andritsos L., Jones J.A., Flynn J.M. (2012). ER stress and autophagy: New discoveries in the mechanism of action and drug resistance of the cyclin-dependent kinase inhibitor flavopiridol. Blood.

[87] Yeh Y.Y., Chen R., Hessler J., Mahoney E., Lehman A.M., Heerema N.A., Grever M.R., Plunkett W., Byrd J.C., Johnson A.J. (2015). Up-regulation of CDK9 kinase activity and Mcl-1 stability contributes to the acquired resistance to cyclin-dependent kinase inhibitors in leukemia. Oncotarget.

[88] Decker R.H., Wang S., Dai Y., Dent P., Grant S. (2002). Loss of the BCL-2 phosphorylation loop domain is required to protect human myeloid leukemia cells from flavopiridol-mediated mitochondrial damage and apoptosis. Cancer Biol. Ther..

[89] Gorlick R., Kolb E.A., Houghton P.J., Morton C.L., Neale G., Keir S.T., Carol H., Lock R., Phelps D., Kang M.H. (2012). Initial testing (stage 1) of the cyclin dependent kinase inhibitor SCH 727965 (dinaciclib) by the pediatric preclinical testing program. Pediatr. Blood Cancer.

[90] Moharram S.A., Shah K., Khanum F., Marhäll A., Gazi M., Kazi J.U. (2017). Efficacy of the CDK inhibitor dinaciclib in vitro and in vivo in T-cell acute lymphoblastic leukemia. Cancer Lett..

[91] Gojo I., Sadowska M., Walker A., Feldman E.J., Iyer S.P., Baer M.R., Sausville E.A., Lapidus R.G., Zhang D., Zhu Y. (2013). Clinical and laboratory studies of the novel cyclin-dependent kinase inhibitor dinaciclib (SCH 727965) in acute leukemias. Cancer Chemother. Pharmacol..

[92] Baker A., Gregory G.P., Verbrugge I., Kats L., Hilton J.J., Vidacs E., Lee E.M., Lock R.B., Zuber J., Shortt J. (2016). The CDK9 inhibitor dinaciclib exerts potent apoptotic and antitumor effects in preclinical models of MLL-rearranged acute myeloid Leukemia. Cancer Res..

[93] Johnson A.J., Yeh Y.-Y., Smith L.L., Wagner A.J., Hessler J., Gupta S., Flynn J., Jones J., Zhang X., Bannerji R. (2012). The novel cyclin-dependent kinase inhibitor dinaciclib (SCH727965) promotes apoptosis and abrogates microenvironmental cytokine protection in chronic lymphocytic leukemia cells. Leukemia.

[94] Chen Y., Germano S., Clements C., Samuel J., Shelmani G., Jayne S., Dyer M.J.S., Macip S. (2016). Pro-survival signal inhibition by CDK inhibitor dinaciclib in Chronic Lymphocytic Leukaemia. Br. J. Haematol..

[95] Fabre C., Gobbi M., Ezzili C., Zoubir M., Sablin M.-P., Small K., Im E., Shinwari N., Zhang D., Zhou H. (2014). Clinical study of the novel cyclin-dependent kinase inhibitor dinaciclib in combination with rituximab in relapsed/refractory chronic lymphocytic leukemia patients. Cancer Chemother. Pharmacol..

[96] Ofatumumab and Dinaciclib in Treating Patients With Relapsed or Refractory Chronic Lymphocytic Leukemia, Small Lymphocytic Lymphoma, or B-Cell Prolymphocytic Leukemia—Study Results—ClinicalTrials.gov. https://clinicaltrials.gov/ct2/show/NCT01515176.

[97] Höring E., Montraveta A., Heine S., Kleih M., Schaaf L., Vöhringer M.C., Esteve-Arenys A., Roué G., Colomer D., Campo E. (2017). Dual targeting of MCL1 and NOXA as effective strategy for treatment of mantle cell lymphoma. Br. J. Haematol..

[98] Nemunaitis J.J., Small K.A., Kirschmeier P., Zhang D., Zhu Y., Jou Y.M., Statkevich P., Yao S.L., Bannerji R. (2013). A first-in-human, phase 1, dose-escalation study of dinaciclib, a novel cyclin-dependent kinase inhibitor, administered weekly in subjects with advanced malignancies. J. Transl. Med..

[99] Mita M.M., Mita A.C., Moseley J.L., Poon J., Small K.A., Jou Y.M., Kirschmeier P., Zhang D., Zhu Y., Statkevich P. (2017). Phase 1 safety, pharmacokinetic and pharmacodynamic study of the cyclin-dependent kinase inhibitor dinaciclib administered every three weeks in patients with advanced malignancies. Br. J. Cancer.

[100] Tang H., Xu L., Liang X., Gao G. (2018). Low dose dinaciclib enhances doxorubicin-induced senescence in myeloma RPMI8226 cells by transformation of the p21 and p16 pathways. Oncol. Lett..

[101] Alagpulinsa D.A., Ayyadevara S., Yaccoby S., Reis R.J.S. (2016). A Cyclin-dependent kinase inhibitor, dinaciclib, impairs homologous recombination and sensitizes multiple myeloma cells to PARP inhibition. Mol. Cancer Ther..

[102] Kumar S.K., LaPlant B., Chng W.J., Zonder J., Callander N., Fonseca R., Fruth B., Roy V., Erlichman C., Stewart A.K. (2015). Dinaciclib, a novel CDK inhibitor, demonstrates encouraging single-agent activity in patients with relapsed multiple myeloma. Blood.

[103] Santo L., Vallet S., Hideshima T., Cirstea D., Ikeda H., Pozzi S., Patel K., Okawa Y., Gorgun G., Perrone G. (2010). AT7519, A novel small molecule multi-cyclin-dependent kinase inhibitor, induces apoptosis in multiple myeloma via GSK-3beta activation and RNA polymerase II inhibition. Oncogene.

[104] Squires M.S., Cooke L., Lock V., Qi W., Lewis E.J., Thompson N.T., Lyons J.F., Mahadevan D. (2010). AT7519, a cyclin-dependent kinase inhibitor, exerts its effects by transcriptional inhibition in leukemia cell lines and patient samples. Mol. Cancer Ther..

[105] Chen E.X., Hotte S., Hirte H., Siu L.L., Lyons J., Squires M., Lovell S., Turner S., McIntosh L., Seymour L. (2014). A Phase I study of cyclin-dependent kinase inhibitor, AT7519, in patients with advanced cancer: NCIC Clinical Trials Group IND 177. Br. J. Cancer.

[106] Seftel M.D., Kuruvilla J., Kouroukis T., Banerji V., Fraser G., Crump M., Kumar R., Chalchal H.I., Salim M., Laister R.C. (2017). The CDK inhibitor AT7519M in patients with relapsed or refractory chronic lymphocytic leukemia (CLL) and mantle cell lymphoma. A Phase II study of the Canadian Cancer Trials Group. Leuk. Lymphoma.

[107] Paiva C., Godbersen J.C., Soderquist R.S., Rowland T., Kilmarx S., Spurgeon S.E., Brown J.R., Srinivasa S.P., Danilov A.V. (2015). Cyclin-dependent kinase inhibitor P1446A induces apoptosis in a JNK/p38 MAPK-dependent manner in chronic lymphocytic leukemia B-cells. PLoS ONE.

[108] Luedtke D.A., Su Y., Ma J., Li X., Buck S.A., Edwards H., Polin L., Kushner J., Dzinic S.H., White K. (2020). Inhibition of CDK9 by voruciclib synergistically enhances cell death induced by the Bcl-2 selective inhibitor venetoclax in preclinical models of acute myeloid leukemia. Signal Transduct. Target. Ther..

[109] Joshi K.S., Rathos M.J., Joshi R.D., Sivakumar M., Mascarenhas M., Kamble S., Lal B., Sharma S. (2007). In vitro antitumor properties of a novel cyclin-dependent kinase inhibitor, P276-00. Mol. Cancer Ther..

[110] Raje N., Hideshima T., Mukherjee S., Raab M., Vallet S., Chhetri S., Cirstea D., Pozzi S., Mitsiades C., Rooney M. (2009). Preclinical activity of P276-00, a novel small-molecule cyclin-dependent kinase inhibitor in the therapy of multiple myeloma. Leukemia.

[111] Shirsath N.P., Manohar S.M., Joshi K.S. (2012). P276-00, a cyclin-dependent kinase inhibitor, modulates cell cycle and induces apoptosis in vitro and in vivo in mantle cell lymphoma cell lines. Mol. Cancer.

[112] Cassaday R.D., Goy A., Advani S., Chawla P., Nachankar R., Gandhi M., Gopal A.K. (2015). A phase II, single-arm, open-label, multicenter study to evaluate the efficacy and safety of P276-00, a cyclin-dependent kinase inhibitor, in patients with relapsed or refractory mantle cell lymphoma. Clin. Lymphoma Myeloma Leuk..

[113] Bahleda R., Grilley-Olson J.E., Govindan R., Barlesi F., Greillier L., Perol M., Ray-Coquard I., Strumberg D., Schultheis B., Dy G.K. (2017). Phase i dose-escalation studies of roniciclib, a pan-cyclin-dependent kinase inhibitor, in advanced malignancies. Br. J. Cancer.

[114] Kolar Z., Flavell J.R., Ehrmann J., Rihakova P., Macak J., Lowe D., Cracker J., Vojtesek B., Young L.S., Murray P.G. (2000). Apoptosis of malignant cells in Hodgkin’s disease is related to expression of the cdk inhibitor p27KIP1. J. Pathol..

[115] Sánchez-Aguilera A., Delgado J., Camacho F.I., Sánchez-Beato M., Sánchez L., Montalbán C., Fresno M.F., Martín C., Piris M.A., García J.F. (2004). Silencing of the p18INK4c gene by promoter hypermethylation in Reed-Sternberg cells in Hodgkin lymphomas. Blood.

[116] García J.F., Villuendas R., Algara P., Sáez A.I., Sánchez-Verde L., Martínez-Montero J.C., Martínez P., Piris M.A. (1999). Loss of p16 protein expression associated with methylation of the p16INK4A gene is a frequent finding in Hodgkin’s disease. Lab. Invest..

[117] Keegan K., Li C., Li Z., Ma J., Ragains M., Coberly S., Hollenback D., Eksterowicz J., Liang L., Weidner M. (2014). Preclinical evaluation of AMG 925, a FLT3/CDK4 dual kinase inhibitor for treating acute myeloid leukemia. Mol. Cancer Ther..

[118] Wang Y., Zhi Y., Jin Q., Lu S., Lin G., Yuan H., Yang T., Wang Z., Yao C., Ling J. (2018). Discovery of 4-((7H-Pyrrolo[2,3-d]pyrimidin-4-yl)amino)-N-(4-((4-methylpiperazin-1-yl)methyl)phenyl)-1H-pyrazole-3-carboxamide (FN-1501), an FLT3- and CDK-Kinase Inhibitor with Potentially High Efficiency against Acute Myelocytic Leukemia. J. Med. Chem..

[119] William A.D., Lee A.C.H., Goh K.C., Blanchard S., Poulsen A., Teo E.L., Nagaraj H., Lee C.P., Wang H., Williams M. (2012). Discovery of kinase spectrum selective macrocycle (16E)-14-methyl-20-oxa-5, 7,14,26-tetraazatetracyclo[19.3.1.1(2,6).1(8,12)]heptacosa-1(25),2(26),3,5,8(27),9,11,16,21,23-decaene (SB1317/TG02), a potent inhibitor of cyclin dependent kinases (CDKs), Janus. J. Med. Chem..

[120] Goh K.C., Novotny-Diermayr V., Hart S., Ong L.C., Loh Y.K., Cheong A., Tan Y.C., Hu C., Jayaraman R., William A.D. (2012). TG02, a novel oral multi-kinase inhibitor of CDKs, JAK2 and FLT3 with potent anti-leukemic properties. Leukemia.

[121] Pallis M., Abdul-Aziz A., Burrows F., Seedhouse C., Grundy M., Russell N. (2012). The multi-kinase inhibitor TG02 overcomes signalling activation by survival factors to deplete MCL1 and XIAP and induce cell death in primary acute myeloid leukaemia cells. Br. J. Haematol..

[122] Álvarez-Fernández S., Ortiz-Ruiz M.J., Parrott T., Zaknoen S., Ocio E.M., San Miguel J., Burrows F.J., Esparís-Ogando A., Pandiella A. (2013). Potent antimyeloma activity of a novel ERK5/CDK inhibitor. Clin. Cancer Res..

[123] Cirstea D., Hideshima T., Santo L., Eda H., Mishima Y., Nemani N., Hu Y., Mimura N., Cottini F., Gorgun G. (2013). Small-molecule multi-targeted kinase inhibitor RGB-286638 triggers P53-dependent and-independent anti-multiple myeloma activity through inhibition of transcriptional CDKs. Leukemia.

[124] Divakar S.K.A., Ramana Reddy M.V., Cosenza S.C., Baker S.J., Perumal D., Antonelli A.C., Brody J., Akula B., Parekh S., Premkumar Reddy E. (2016). Dual inhibition of CDK_4_/Rb and PI3K/AKT/mTOR pathways by ON_123300_ induces synthetic lethality in mantle cell lymphomas. Leukemia.

[125] Padgaonkar A., Rechkoblit O., Carpio R.V.D., Pallela V., Subbaiah V., Cosenza S.C., Baker S.J., Reddy M.R., Aggarwal A., Reddy E.P. (2018). Targeting protein kinase CK_2_ and CDK_4/6_ pathways with a multi-kinase inhibitor ON_108110_ suppresses pro-survival signaling and growth in mantle cell lymphoma and T-Acute lymphoblastic leukemia. Oncotarget.

[126] De Dominici M., Porazzi P., Xiao Y., Chao A., Tang H., Kumar G., Fortina P., Spinelli O., Rambaldi A., Peterson L.F. (2020). Selective inhibition of Ph-positive ALL cell growth through kinase-dependent and independent effects by CDK6-specific PROTACs. Blood.

[127] Sun B., Fiskus W., Qian Y., Rajapakshe K., Raina K., Coleman K.G., Crew A.P., Shen A., Saenz D.T., Mill C.P. (2018). BET protein proteolysis targeting chimera (PROTAC) exerts potent lethal activity against mantle cell lymphoma cells. Leukemia.

[128] Qiu X., Li Y., Yu B., Ren J., Huang H., Wang M., Ding H., Li Z., Wang J., Bian J. (2020). Discovery of selective CDK9 degraders with enhancing antiproliferative activity through PROTAC conversion. Eur. J. Med. Chem..

[129] Scheicher R., Hoelbl-Kovacic A., Bellutti F., Tigan A.S., Prchal-Murphy M., Heller G., Schneckenleithner C., Salazar-Roa M., Zöchbauer-Müller S., Zuber J. (2015). CDK6 as a key regulator of hematopoietic and leukemic stem cell activation. Blood.

[130] Cogle C.R. (2013). Overcoming chronic myeloid leukemia stem cell resistance to imatinib by also targeting JAK_2_. J. Natl. Cancer Inst..

[131] Zhang J., Zhou L., Zhao S., Dicker D.T., El-Deiry W.S. (2017). The CDK4/6 inhibitor palbociclib synergizes with irinotecan to promote colorectal cancer cell death under hypoxia. Cell Cycle.

[132] Doucette K., Lai C., Pohlmann P.R. (2020). Rebound lymphocytosis in a patient with chronic lymphocytic leukemia after cessation of a CDK 4/6 inhibitor for concomitant breast cancer. Breast J..

