# Supplementary material for: Cyclin-Dependent Kinase Inhibitors in Hematological Malignancies—Current Understanding, (Pre-)Clinical Application and Promising Approaches
Source: Cancers (Basel). 2021 May 20;13(10):2497. doi: 10.3390/cancers13102497 (PMC8161389; doi:10.3390/cancers13102497)
Supplement: Supplementary file 1 [file cancers-13-02497-s001.zip › cancers-1181671-supplementary.pdf]

Table 1. Overview on recent preclinical and clinical application of CDK inhibitors in hematological malignancies as well as potential combination partners showing boosted effects.

CDK inhibitor	Target structure	Entity	Main mechanism	Synergistic effects in combination with…	Study type		Ref.
					pre-clinical	clinical	
**palbociclib**	CDK4/6	AML, B-ALL, CML, CLL, MCL, DLBCL, BL, ALCL, MM	Rb dephosphorylation, G1 arrest/senescence, apoptosis, autophagy	AML: sequential low-dose cytarabine, danusertib, MK-2206<br>ALL: FGFR1 inhibitor PD-173074, imatinib<br>MM: dexamethasone, bortezomid<br>CML: ponatinib<br>MCL: ibrutinib, PI3K inhibitors (re-sensitization)<br>MM: dexamethasone, bortezomib	✓	✓	14, 17, 20-22, 25-30, 32, 33, 35-40, 42-45
**ribociclib**	CDK4/6	AML, ALL	G1 arrest/senescence/apoptosis	ALL: dexamethasone, everolimus	✓	✓	48, 49, 51
**abemaciclib**	CDK4/6	AML, MCL, DLBCL, MM	G1 arrest/senescence, autophagy, decreased MAPK and AKT pathway signaling				54-58
**lerociclib**	CDK4/6	AML, ALL, CML, BL	decreased Rb phosphorylation, G1 arrest		✓		59, 83
**roniciclib**	Pan-CDKs (CDK1/2/3/4/7/9)	CLL, HL			✓	✓	113
**flavopiridol**	Pan-CDKs (CDK1/2/4/6/7/9)	AML, ALL, CML, CLL, DLBCL	cell cycle arrest , MCL-1, BIM and NOXA reduction, ER stress-mediated death	AML/MM: venetoclax/ obatoclax<br>CML: pyrrolo-1,5-benzoxazepine compounds	✓	✓	79, 80, 82, 83, 86, 113
**voruciclib**	Pan-CDKs (CDK1/2/4/5/6/8/9)	AML, CLL, FL, MCL, DLBCL, AML	Mcl-1 and c-Myc downregulation, caspase-3 and PARP-dependent apoptosis, JNK and p38	AML: venetoclax	✓	✓	61, 107

			phosphorylation, NOXA induction				
**THZ1**	CDK7/12/13	AML	apoptosis	MM: venetoclax or carfilzomib	✓	✓	75, 76
**SY-1365**	CDK7	AML	apoptosis	AML: venetoclax	✓		77
**YKL-06-101**	CDK8, mTOR	AML, ALL	growth inhibition				73
**SEL120-34A**	CDK8/19	AML, MDS	growth inhibition		✓	✓	74
**atuveciclib**	CDK9, PTEFb, GSK3	AML, MCL, BL	apoptosis	AML: venetoclax<br>MCL: metformin	✓		66, 67, 128
**AZD4573**	CDK9	AML, ALL, MCL, DLBCL, BL, MM	Caspase activation, PARP cleavage, MCL-1 suppression, XIAP down-regulation	AML/ALL/ DLBCL: venetoclax<br>DLBCL: EZH2 inhibitors EPZ6438 and GSK126	✓	✓	62
**CDKI-73**	CDK1, 2, 9	AML, ALL, CLL, DLBCL	MCL-1 down-regulation, apoptosis induction	AML: venetoclax<br>CLL: fludarabine	✓	✓	65
**A-1592668**	CDK9	AML, NHL	reduced RNA polymerase II activity, loss of MCL-1 protein, apoptosis	AML/NHL: venetoclax	✓	✓	70
**LDC526**	PTEFb/CDK9	CLL	Inhibited proliferation		✓		68
**dinaciclib**	CDK1/2/5/9	MLL-rearranged AML, B- and T-ALL, CLL, MCL, MM	Mcl-1 downregulation, senescence	CLL: ibrutinib, venetoclax, rituximab/ ofatumumab<br>MCL: orlistat<br>MM: doxorubicin, PARP inhibitor ABT-888	✓	✓	47, 89, 91-93, 95-98, 100-102, 106
**AT7519 (alternative name: AT7519M)**	CDK1/2/4/5/9, GSK3β	CLL, MCL, MM	apoptosis, inhibition of RNA polymerase II and GSK3b phosphorylation		✓	✓	103, 104, 106
**P276-00**	CDK1/4/9	AML, MM	cell cycle arrest, caspase activation, Rb dephosphorylation, cyclin downregulation	MM: bortezomib	✓	✓	109-112
**TG02**	CDK1/2/7/ 9, JAK2, FLT3	AML, MM	G1 arrest, ERK5 blockade, intrinsic and extrinsic apoptosis induction	MM: dexamethasone, melphalan, bortezomib lenalidomide	✓		119-123

AML – acute myeloid leukemia, ALCL - Anaplastic large cell lymphoma, B-ALL – B- acute lymphoblastic leukemia, BL – burkitt lymphoma, CLL - chronic lymphoblastic leukemia, CML – chronic myeloid leukemia, DLBCL - Diffuse large B cell lymphoma, FL – follicular lymphoma, MCL - Mantle cell lymphoma, MDS - Myelodysplastic syndrome, MM – multiple myeloma, NHL – non-Hodgkin lymphoma, Rb – retinoblastoma
